# The association of family food security and depression in mothers having primary school children in Ray-Iran

**DOI:** 10.1186/2251-6581-13-65

**Published:** 2014-05-28

**Authors:** Moloud Payab, Ahmad-reza Dorosty Motlagh, Mohammadreza Eshraghian, Reza Rostami, Fereydoun Siassi

**Affiliations:** 1 Obesity and Eating Habits Research Center, Endocrinology and Metabolism Molecular -Cellular Sciences Institute, Tehran University of Medical Sciences, Tehran, Iran; 2 Head of Community Nutrition, School of Nutrition Science and Dietetics, Tehran University of Medical Sciences, Tehran, Iran; 3 School of Public Health & Institute of Public Health Researches, Tehran University of Medical Sciences, Tehran, Iran; 4 Institute for Psychology and Educational Sciences, University of Tehran, Tehran, Iran; 5 School of Nutrition Science and Dietetics, Tehran University of Medical Sciences, Tehran, Iran

**Keywords:** Food security, Depression, Mother

## Abstract

**Background:**

As a major public health problem, food insecurity has adverse social and psychological effects, in addition to the impact on public health. This study aimed to determine the association of household food security and depression in mothers with primary school children in Ray County.

**Methods:**

This descriptive, analytical cross-sectional study was conducted on 430 mothers with primary school children in the spring 2010. During a two-stage cluster sampling, an 18-items food security questionnaire (USDA) and the Beck depression inventory were completed via interviewing mothers. Chi-squared test, one-way analysis of variance, simple regression and stepwise multiple regression were used to describe and analyze data, and to identify related factors using SPSS-16 software.

**Results:**

The prevalence of food insecurity and depression in mothers were 50.2% and 51.4% respectively. Also 34.6% of mothers in the "food secure" group were depressed and 77.8% in "food insecure with hunger" group were depressed and this difference was statistically significant. Twelve out of the 20 examined variables (age, family size, number of children, economic status, home ownership, employment households, educational level of the mother and also the head of household, height, energy intake, and carbohydrate and protein intakes) were significantly associated with food security and depression. Food insecurity and depression in mothers with primary school children in Ray County showed a significant positive correlation (P < 0.001).

**Conclusion:**

The prevalence of household food insecurity and depression in the studied population were high. Since there is a significant relationship between food insecurity and depression, more attention must be paid to this group.

## Introduction

Depression is the most common mood disorder that all people may experience it as a short-term or long-term situation in their lives. Changes in appetite, weight, sleep and activity, lack of energy, feeling of guilt, difficulty in decision-making, lack of motivation and lack of pleasure are key symptoms of depression [1,2].

According to the World Health Organization Report 2000, depression is ranked fourth in diseases across the world, and is considered the most common cause of disability. Furthermore, it is predicted that in 2020, this disorder will be ranked second in the world in terms of disease burden [3]. Also, according to the World Health Organization Report, 121 million people around the world are suffering from depression [3]. In Iran, the prevalence of depression has been estimated by Noorbala et al., as high as 3.8% and is at the top of mental disorders [1]. In another study, Kaviani et al. showed that the rate of depression in Tehran (Iran capital) is 12.16% and 8.47% in women and men, respectively [4].

The prevalence of depression is higher in women than men and often occurs in women between 18–24 years of age, especially those having children. Given that women consist of half the population of each community, if they are employed, their job performance will be affected in housewives, depression will decrease their performance in home and will increase family problems and conflicts and will impose high costs on the society [5].

Food and nutrition are of basic needs of human society which supply lies in the food security category. Food security is defined as: the access of all people to always and enough food, in order to have an active and healthy lifestyle, which includes: 1) availability of nutritionally adequate and safe food, 2) ability and confidence to acquire acceptable foods in a way that is acceptable to the community [6]. The first report on the food security situation in America was presented in 1995 using the census center data. According to the results of that study, about 11.9% of households were food insecure [7]. Research showed that in 2004 more than 800 million people around the world were suffering from starvation [8]. The prevalence of food insecurity in American households was reported 14.7% in 2009 [9]. A study conducted in Iran (1998) also revealed that 20% of people have no economic access to abdominal fullness and about 50% have problems for the supply of cell fullness. In other words, one fifth of people had a lack of energy and half of them were deficient in micronutrients [10]. In another study, the overall prevalence of food insecurity in households was reported 44% in Shiraz in 2008 [11].

Food insecurity and hunger have adverse social and psychological effects (e.g. depression), in addition to the impact on health [12]. Casey et al. conducted a study aiming to examine the relationship between maternal depression and food security and health status of children in 5 states of U.S.A. This study was conducted on 5,306 mothers with children under 3 years old. The results of this study showed that there was a positive relationship between household food insecurity and maternal depression. In depressed mothers, 32.9% were food secure and 67.1% were food insecure [13].

The sample in the Huddleston-Casas’s study was composed of 413 mothers with at least one child under 13 years old. The results showed that there is a bidirectional cause and effect association between food insecurity and depression [14].

Meanwhile, mothers are one of the most vulnerable groups in the society. Give the mother's role as head of household, and that mothers usually have more detailed information about the food security status of the household, and this group is more likely to be depressed, thus, it is necessary to study the relationship between food security and maternal depression.

## Materials and methods

### Research methodology

This study is a descriptive, analytical cross sectional. This study was conducted on 430 mothers with primary school children living in Ray County in 2010.

### Sample size and sampling method

The population of this study was composed of all mothers with primary school children in Ray County in 2010. The sampling method was two-stage cluster.

A pilot study was designed and implemented to determine the sample size. According to the pilot study, 38% of mothers (93 mothers) who were randomly selected, fell in the food secure group. Also, the percentage of depressed mothers among the food insecure and food secure mothers was 63% and 48%, respectively. Thus, based on the statistical formula, it was necessary to examine 430 mothers so that their depression difference could be found.

The number of schools in Ray County was specified by referring to (questioned from) the Ray Education Center. Forty three elementary schools were selected randomly from 102 schools in Ray. After coordination with school administrators, about 10 children were randomly selected from each school and their mothers were invited to the schools.

### Data collection methods

After a complete explanation about the objectives of the study, informed consent was obtained from mothers.

Weight evaluation: Mothers height status was measured by the Seca stadiometer with a precision of 0.1 cm while the person was attached to the wall without shoes, looking forward. Mother’s weight was measured by a Seca flat digital scale with a precision of 0.1 kg, while the person was wearing minimal clothing and no shoes. Their body mass index (BMI) was calculated by dividing weight (kg) by height^2^ (m^2^). Mother’s weight status was evaluated according to the WHO standard [15].

### Food security assessment

Food security status was evaluated by the USDA (US Department of Agriculture) questionnaire, which has been used in the U.S. Current Population Survey annually since 1995 [6]. This 18-statement questionnaire which examined household food security status in the last 12 months was completed by interviewing mothers. The studied mothers were divided into four groups based on the scores of the questionnaire: Food secure, food insecure without hunger, Food insecure with moderate hunger and food insecure with severe hunger (Table 1). Then, the two groups of "food insecure with moderate hunger" and "food insecure with severe hunger" were merged together and formed the food insecure with hunger group. the validity of this questionnaire has been evaluated in previous study [16].

**Table 1:** Classification of the household food security status based on scores

**Food security status**	**Number of positive responses**
Food secure	0-2
Food insecure without hunger	3-7
Food insecure with moderate hunger	8-12
Food insecure with severe hunger	13-18

### Depression assessment

Maternal depression was assessed by the Beck Depression Inventory. In this questionnaire, there are several groups of questions and each question represents a state in the individual. The respondent should select the option that best represents his/her current feeling (i.e. just what he/she feels at the time of completing the questionnaire). The studied mothers were divided into six groups based on the scores of the questionnaire: Normal, a little depressed, need to consult with a psychiatrist, moderately depressed, severely depressed, and excessively depressed (Table 2). Then, they were classified into 3 groups of normal, mild and moderate, and severe depression.

**Table 2:** Classification of the maternal depression status based on scores

**Depression status**	**Total score**
Normal	1-10
A little depressed	11-13
Need to consult with a psychiatrist	17-20
Moderately depressed	21-30
Severely depressed	31-40
Excessively depressed	> 40

### 24-hour dietary recall

One method to examine a person's nutritional status is 24-hour dietary recall which is of retrospective data type. In this method, person is asked about appetite status, consumption of vitamins and minerals or medicines and the eating speed. Also, he/she is asked to recall and report all meals including food and beverages consumed in the past 24 hours. In this study, 24-hour dietary recall was taken from mothers two times (the first in a typical day in the middle of the week and the second on a holiday). Then, these values were entered in Dorosty Food Processor for Windows software (DFPW-2.1), and the amounts of energy, carbohydrate, protein and fat intake were calculated.

### Assessment of socio-economic and demographic status

Demographic characteristics (including maternal age, family size, number of children, and social and economic characteristics) were collected by the general information questionnaire. These characteristics were as follows: education and occupational status of mother and head of household, residential property ownership status and having living facilities. About living facilities, mothers were asked that how many items of these 9 items they have (furniture, handcraft carpet, refrigerator, washing machine, dishwasher, microwave, computer, car, and home). Having less than or equal to 3 items was considered as low economic status, 4 to 6 items as moderate economic situation and 7 to 9 items as good economic status [11]. About ownership of house, mothers were asked to select one of the options of private house, rent or mortgage, and living with parents or relatives and others.

### Statistical analysis method

After collecting the data, it was statistically analyzed using SPSS-16 software. The classes of food security and depression for each household were determined according to the obtained score. The relationship between qualitative variables and classes of household food security and depression were evaluated with Chi-squared test. Mean and standard deviation were calculated for displaying the quantitative variables' results. The simple regression method was used to assess the relationship between food security status and depression. Also, the multiple regression method was used to determine the variables that were most statistically significant with food security status and depression. Then, variables were entered into the model step by step forward.

The food consumption was converted into food material and its value was calculated (in grams) and coded. By inserting these values in the Nutritionist 4 application, the values for energy, carbohydrate, protein and fat were calculated.

## Results

In this study, 430 mothers were studied. The prevalence of food insecurity among the studied mothers was 50.2%. So that the values of food insecurity without hunger, with moderate and severe hunger were estimated 31.4, 15.3 and 3.5 percent, respectively. Due to the low percentage of food insecurity with severe hunger, in other analyzes, the two groups of food insecurity with moderate and severe hunger were combined (18.8%). Among the studied variables, there was a significant relationship between 11 variables (age, family size, number of children, economic status, occupation of household head, education level of mother and head of households, height, energy intake, carbohydrate and protein intake) and food security (Tables 3 and 4).

**Table 3:** Mean and standard deviation (SD) of anthropometric characteristics, daily intake of energy and macronutrients and demographic variables examined in accordance with food security status and result of ANOVA in order to compare them amongst mothers studied

**ANOVA P-Value**	**Total n = 430**	**Food insecurity with hunger n = 81**	**Food insecurity without hunger n = 135**	**Food secure n = 214**	**Variables**
0.001	34.7 ± 5.3	36.3 ± 5.6	35.2 ± 4.9	33.8 ± 5.2	Age (years)
<0.001	4.0 ± 0.9	4.4 ± 1.1	4.0 ± 0.9	3.9 ± 0.9	Family size
<0.001	2.0 ± 0.9	2.4 ± 1.2	2.0 ± 0.9	1.8 ± 0.8	Number of children
0.53	70.6 ± 13.1	70.6 ± 13.2	71.6 ± 13.0	69.9 ± 13.2	Weight
0.035	158.6 ± 5.8	157.2 ± 5.3	158.7 ± 5.5	159.1 ± 6.0	Height
0.177	28.0 ± 5.0	28.6 ± 5.1	28.4 ± 5.0	27.6 ± 4.9	BMI
0.036	2074.8 ± 720.4	1896.4 ± 718.3	2076.5 ± 644.4	2142.8 ± 756.9	Energy (Kcal)
0.046	317.8 ± 120.1	288.1 ± 127.8	322.9 ± 108.8	326.2 ± 122.5	Carbohydrate (gr)
0.012	61.1 ± 21.8	53.7 ± 19.3	62.1 ± 18.9	63.2 ± 23.8	Protein (gr)
0.352	71.4 ± 32.2	69.3 ± 40.4	68.5 ± 26.3	74 ± 31.9	Fat (gr)

Data are set as the mean ± SD.

**Table 4:** Correlation between food security status and qualitative variables

**Variables**	**Food secure**	**Food insecure without hungry**	**Food insecure with hungry**	**Total**	**χ2 test/P-value**
**Marital status**					0.213
Married	209(50.2)	131(31.5)	76(18.3)	416(100)	
Widow	5(35.7)	4(28.6)	5(35.7)	14(100)	
Total	214(49.8)	135(31.4)	81(18.8)	430(100)	
**Occupational status**					
Housekeepers	204(49.5)	130(31.6)	78(18.9)	412(100)	0.882
Employed	10(55.6)	5(27.8)	3(16.7)	18(100)	
**Occupational status of the head of household (n = 415)**					
Worker	34(36.2)	28(29.8)	32(34.0)	16(100)	<0.001
Government employee	77(59.7)	43(33.3)	9(7.0)	129(100)	
Self-employed	86(53.8)	51(31.9)	23(14.4)	160(100)	
Unemployed and retired	11(34.6)	9(28.1)	12(37.3)	32(100)	
**Education level**					
Illiterate/elementary	19(26.0)	25(34.2)	29(39.7)	73(100)	<0.001
Secondary school/high school	167(52.4)	101(31.7)	15(16.0)	310(100)	
University	28(73.7)	9(23.7)	1(2.6)	38(100)	
**Education level of the head of household**					
Illiterate/elementary	15(22.4)	24(35.8)	28(41.8)	67(100)	<0.001
Secondary school/high school	141(52.0)	89(32.8)	41(15.1)	271(100)	
University	53(67.9)	18(23.1)	7(9.0)	78(100)	
**Socioeconomic status (n = 428)**					
Low	47(34.1)	45(32.6)	46(33.3)	138(100)	<0.001
Middle	128(52.7)	84(34.6)	31(12.8)	243(100)	
High	39(83.0)	6(12.8)	2(4.3)	47(100)	

**Figure 1:**
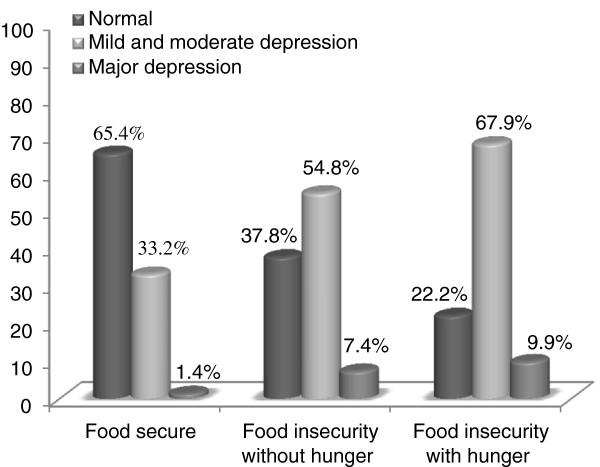
Distribution of samples according to food security and depression status.

Also, the prevalence of depression in mothers was 51.4%: 46.5% had mild and moderate depression and 4.9% had severe depression. Among the examined variables, 12 variables (age, family size, number of children, marital status, economic status, education level of the mother and head of household, occupation of mother and head of household, energy intake, carbohydrate and protein intake) were significantly associated with depression [17].In the "food secure" group, 34.6% of mothers were depressed and in the "food insecure with hunger" group, 77.8% were depressed. Food insecurity and depression in mothers with primary school children in Ray County showed a significant positive correlation (Figure 1).

According to Table 5, only the variables of being worker, clerk and retired of head of households could affect the relationship between depression scores and food security scores.

**Table 5:** Correlation between depression rating, food security ratings and other variables

**Variables**	**β ± SE**	**P-value**
Constant value	33.0 ± 61.77	0.593
Age	-0.11 ± 0.1	0.281
Family size	0.21 ± 0.68	0.76
Number of children	0.89 ± 0.78	0.252
Socio-economic level	-0.37 ± 0.31	0.235
Weight	0.18 ± 0.43	0.681
Height	-0.1 ± 0.39	0.792
BMI	-0.4 ± 1.07	0.707
Education level of mother		
Illiterate (basic group)	-	
Diploma	1.86 ± 1.4	0.183
University	-0.34 ± 2.03	0.865
Education level of the head of household		
Illiterate (basic group)	-	
Diploma	-1.37 ± 1.45	0.346
University	-0.18 ± 1.82	0.92
**Occupational status of mother**		
Housekeepers (basic group)	-	
Employment	2.19 ± 2.05	0.285
**Occupational status of the head of household**		
Unemployed (basic group)	-	
Worker	-4.33 ± 2.25	0.027
Government employee	-4.06 ± 2.29	0.038
Self-employed	-2.8 ± 2.2	0.205
Retired	-6.08 ± 3.02	0.022
**Marital status**		
Being married (basic group)	-	
Being widowed	0.72 ± 3.21	0.823
Food security score	0.87 ± 0.12	<0.001

## Discussion

The prevalence of depression in the examined mothers was 51.4% [17]. In a study was conducted in Malatya, Turkey which reported the prevalence of depression in women before and after menopause 41.8% [18]. The prevalence of depression in this study was higher than all of the mentioned studies. This difference could be attributed to the different population studied.

The results of our study showed that 50.2% of households in Ray County had mild to severe food insecure [17]. In a study conducted on households in Shiraz, the prevalence of food insecurity was 44% [11]. In a study on Yazdi households with 6–11 year-old children, Karam Soltani et al. reported the prevalence of food insecurity 30.5% [19]. Also, in a study on the residents of Asadabadi district in Tabriz, the prevalence of food insecurity was reported 36.3% [20]. The prevalence of food insecurity in the United States fluctuated between 10.1% and 14.7% during 1995–2009 [9]. But the prevalence of food insecurity was higher in the present study.

In our study, food insecurity had a significant positive correlation with age and family size (P = 0.001). In studies by Ramesh et al. on families in Shiraz [11], by Mohammad et al. on adolescents and their mothers [21], and by Huddleston-Casas [14], there was no significant relationship between food insecurity and age. But in the study by Mohammadi Nasrabadi, there was a significant positive relationship between food insecurity and age [22]. Also, in a study by Kaiser et al. on women in California, a significant negative relationship was found between food insecurity and age [23]. In studies by Dastgiri et al., Mohammadzadeh et al. and Chaput et al., there was a significant positive relationship between the prevalence of food insecurity and family size [20,21,24]. Ramesh et al., Che and Chen, Willows et al., and Foley et al., showed that here was a significant positive relationship between family size (number of children) and food insecurity [11,25-27].

In this study, this significant relationship may be justifies that with the increase in family size, the need for food increases. As a result, the size and number of meals can also be reduced and food insecurity to emerge.

Also, in this study, the prevalence of food insecurity in households of the first tecile (low socioeconomic status) was higher which is consistent with the results of the studies by Ramesh et al. and Mohammad Zadeh et al. [11,21]. The prevalence of food insecurity in households with income below the poverty level was also higher in the United States of America [9]. Households with higher incomes and better economic conditions have more choices in food selection and can spend a greater percentage of their income on food.

In this study, there was a significant relationship between food insecurity and job status of head of household but there was a significant relationship between food insecurity and job status of mother. Results showed that food security was higher in mothers whose husbands were clerks, and vice versa; also, food insecurity was higher in mothers whose husbands were unemployed. A study by Mohammad Zadeh et al. showed a significant relationship between food insecurity and job status of mother and head of household [21]. In their study, Dastgiri et al. showed that the higher the job status of head of household, the lower the food insecurity (P < 0.001) [20]. In other studies on Canadian households, rural households in Malaysia and women living in California, there was a significant relationship between food insecurity and job status [23,25,28].

There was a significant inverse relationship between food insecurity and education level of mother and head of household. Other studies also showed a significant relationship between food insecurity and education level of mother and head of household [11,21,23,24,28]. Lack of adequate education restricts job opportunity and reduces the ability to earn money. Following the reduction of income, feed costs also are affected. Low education level can also reduce people's knowledge level on nutrition and affect all stages of the basket to the table (shopping, preparation, cooking and consumption) which can lead to the household food insecurity.

The study also showed that there is a significant positive relationship between average daily energy intake (P = 0.036/0), average daily carbohydrates intake (P = 0.046), average daily protein intake (P = 0.012) and food security status which is consistent with the results of the study by Kirkpatrick and Tarasuk [29]. So that average intake in the food secure group was higher than the food insecure group. Reduced energy and nutrients may be due to the lack of access to enough food.

In their study, Rosas et al. showed that average daily energy intake and also average daily carbohydrates intake are higher in the food insecure group. In this group, the consumption of cheaper foods which contain high calorie and low quality is higher (sweet and fatty foods are more affordable) [30].

The results of this study showed that 34.6% of mothers in the "food secure" group and 77.8% in the "food insecure with hunger" group were depressed. Also, there was a significant positive relationship between food insecurity and depression in mothers with primary school children living in Ray (P < 0.001), which is consistent with the results of the study by Kim and Frongillo. According to this study, food insecurity is positively associated with weight gain and depression among the elderly [31]. In a study, Whitaker et al. showed that 15.7% of mothers of children under 3 years old in the "food secure" group, 20.2% of mothers in the "food security margin" group and 28.5% of mothers in the "food insecure" group were depressed [32].

Casey et al. conducted a study on mothers of children under 3 years old who live in the District of Columbia. A significant difference was reported between food insecurity and depression in mothers of children under 3 years old (P < 0.001); 32.9% of mothers in the "food secure" group and 67.1% of mothers in the "food secure" group were depressed [14]. Hadley and Patil also showed that in the Tanzanian rural community, there is a significant positive relationship between food security score and anxiety and depression (001/0 > P) [33]. Huddleston et al. conducted a study on rural low-income mothers which showed that there is a bidirectional cause and effect relationship between food insecurity and depression [14].

In the present study, part of the person's concerns may be due to lack of food supply and lack of access to food. This additional mental burden can be effective on maternal depression.

In interpreting the existing results, some limitations should be considered. One limitation of this study was the 18-statement USDA Household Food Security questionnaire which was not validated in the studied population. Also, other causes of depression were not considered in this studied sample.

It is suggested that in future studies, mothers in all age groups should be considered and the used questionnaire should be previously validated in the studied population.

## Competing interests

The authors have no financial competing interests. The data may disclose prevalence of food insecurity and association of family food security and depression in mothers having primary school children living in Rey, South of Tehran, Iran.

## Authors’ contributions

MP participated in the study design, data acquisition, statistical analysis, and interpretation. AD participated in the study design and interpretation. HZ participated in the data acquisition. ME participated in the statistical analysis. RR acted as psychology advisor in collection and interpretation of data. FS participated in the study design. All authors read and approved the final manuscript.
